# An Efficient Protocol for Deriving Liver Stem Cells from Neonatal Mice: Validating Its Differentiation Potential

**DOI:** 10.1155/2015/219206

**Published:** 2015-10-18

**Authors:** Sugapriya Dhanasekaran, Devilakshmi Sithambaram, Kavitha Govarthanan, Bijesh Kumar Biswal, Rama S. Verma

**Affiliations:** ^1^Stem Cell and Molecular Biology Lab, Department of Biotechnology, Bhupat and Jyoti Mehta School of Biosciences, Indian Institute of Technology Madras, Chennai, Tamil Nadu 600036, India; ^2^Department of Medical Laboratory Sciences (Haematology), College of Applied Medical Sciences, Prince Sattam Bin Abdul-Aziz University, Wadi Ad Dawaser Campus, P.O. Box 54, Riyadh, Saudi Arabia

## Abstract

The success of liver regeneration depends on the availability of suitable cell types and their potential to differentiate into functional hepatocytes. To identify the stem cells which have the ability to differentiate into hepatocytes, we used neonatal liver as source. However, the current protocol for isolating stem cells from liver involves enzymes like collagenase, hyaluronidase exposed for longer duration which limits the success. This results in the keen interest to develop an easy single step enzyme digestion protocol for isolating stem cells from liver for tissue engineering approaches. Thus, the unlimited availability of cell type favors setting up the functional recovery of the damaged liver, ensuring ahead success towards treating liver diseases. We attempted to isolate liver stem derived cells (LDSCs) from mouse neonatal liver using single step minimal exposure to enzyme followed by *in vitro* culturing. The cells isolated were characterized for stem cell markers and subjected to lineage differentiation. Further, LDSCs were induced to hepatocyte differentiation and validated with hepatocyte markers. Finally, we developed a reproducible, efficient protocol for isolation of LDSCs with functional hepatocytes differentiation potential, which further can be used as *in vitro* model system for assessing drug toxicity assays in various preclinical trials.

## 1. Introduction

The ability to isolate and expand liver-derived stem cells (LDSCs) is a crucial step towards the success of tissue engineering approaches for liver repair, regeneration for therapeutic purpose, and developing suitable scaffold for liver tissue engineering. Stem cells from the liver tissue can be good candidate cell types of interest in various approaches for regeneration therapy. Liver stem cells having potential to maintain liver homeostasis have considerable therapeutic potential. Hepatic progenitor cells are multipotent stem cells, which exhibit unlimited proliferation giving rise to hepatocytes and bile-duct epithelial cells, residing in the canals of Hering in human and animal livers [1, 2], and* in vivo* terminally differentiated hepatocytes lack the proliferative potential in response to liver injury [3–5]; hence, hepatic progenitor cells may serve as an ideal source for hepatocyte that can be used for transplantation approaches [6–12]. Human fetal liver progenitor cells maintain multipotent capability to differentiate into liver, mesenchymal lineages, and cartilage cells and also have repopulation capacity in a mouse model of liver injury [9].

These hepatocyte progenitor cells are capable of multiple cell divisions and have been identified without a preceding injury to the liver [13]. Earlier reports showed that bipotential clonal cell lines were isolated from adult murine liver [14], and also a report stated that *α*-fetoprotein (AFP) positive cells from adult liver with immature endodermal characteristics were capable of differentiating into both hepatic and biliary cell lineages [15]. Many studies have reported the isolation and purification of liver stem cells directly from the bone marrow using two-step magnetic bead cell sorting procedure [16]. Another report followed the isolation and purification of hepatic stem cell by two-step collagenase perfusion technique, simple gravity enrichment, and immunomagnetic cell sorting methods followed by flow cytometry cell sorting [17]. Earlier study showed that short repetitive trypsinization of heart tissue helps in obtaining a higher yield of viable cardiomyocytes rather than a single digestion for longer period [18].

Various current protocols used enzymes like collagenase, hyaluronidase, or both in combination exposed for a longer duration followed by high speed differential centrifugation using Ficoll density gradient method. Enzyme digestion and LDSCs enrichment are the two crucial steps which determine the success of isolation and culturing of the neonatal liver-derived stem cell* in vitro*. Several techniques, including slow speed centrifugation steps followed after partial collagenase digestion, and Ficoll fractionation, sorted based on the markers expression, have been used to culture neonatal murine LDSCs [11, 14, 19–21]. The enzyme digestion step for dissociating cells from liver tissue is too critical which determines the success of isolating viable neonatal liver stem cells [22]. Although the repetitive digestion with various enzyme combinations gives good yield, long-term enzyme digestion leads to toxicity which limits the success of higher yield in isolating viable cell [23].

In this study, we devised a simple and reproducible protocol for the isolation and culture of LDSCs from neonatal liver and their maintenance in primary cultures that consistently yield long-term liver-derived stem cell cultures. The protocol followed has advantages over the current available protocols, which circumvents the use of any growth supplements, without any collagenase digestion followed by single step enrichment for removing fibroblast contamination.

## 2. Materials and Methods

### 2.1. Animals

Commonly used mouse strains, BALB/c, were used in this protocol. The normal inbred 1- to 2-day-old neonatal mice (male and female) were procured from the King's Institute, Chennai, India. All experiments in this protocol were performed in accordance with the Institutional Animal Ethics Committee (IIT Madras, India, the Committee for the Purpose of Control and Supervision of Experiments on Animals, Government of India). It is preferred to use the neonatal mice (1-2 days old), as our previous observations for cardiomyocytes have shown that younger animals (newborn up to 1-2 days) produce more proliferative cell cultures enrichment [18].

### 2.2. Cell Preparation and Culture

Neonatal mice 1-2-day-old Balb/c pups were euthanized by cervical dislocation. The euthanized pups were rinsed completely in a beaker with 50 mL of 70% (vol/vol) ethanol for 2 min. Then, it was placed in a 100 mm sterile glass dish and incisions were made in the skin of the inguinal region; the muscles were disassociated and the liver was removed aseptically and immediately transferred into ice-cold phosphate-buffered saline (PBS; Ca^2+^ and Mg^2+^ free and 0.1% (vol/vol) penicillin/streptomycin) for 2-3 min. The excised livers were washed again with chilled PBS followed by another wash with sterile ice-cold balanced salt solution (20 mM hydroxyethyl piperazine ethanesulfonic acid, NaOH [pH 7.6], 130 mM NaCl, 1 mM NaH_2_PO_4_, 4 mM glucose, and 3 mM KCl), in which the tissue was kept for 10 min. The livers were then minced with a sterile scalpel blade into small pieces less than or equal to 1 mm^3^ in 2 mL of 0.05% trypsin ethylenediamine tetraacetic acid (EDTA; Invitrogen, Carlsbad, CA; 0.1 mL per liver) and transferred into sterile 15 mL falcon tubes. The hepatic cells were digested by incubating with 0.25% (wt/vol) trypsin 0.02% (wt/vol) EDTA (~1 mL of 0.25% trypsin for every 100 mg of tissue), which was then mixed by intermittent pipetting along with stirring at 37°C in a water bath for 4 min. The cell suspension was allowed to stand for 1 min.

The supernatant containing single cells suspension was collected into a fresh 15 mL falcon tube kept on ice, to which 2 mL Dulbecco's modified Eagle's medium (DMEM)/F12 (1 : 1) medium (Gibco, Carlsbad, CA) supplemented with 20% fetal calf serum (Gibco) was added, and the digestion step was repeated three times. The cell suspension from each digestion was pooled and centrifuged at 3,500 rpm for 10 min at 4°C. The trypsin digestion was then neutralized with complete medium containing FCS. The cell pellet was washed twice in DMEM/F12 (1 : 1) medium supplemented with fetal calf serum (10%) and we finally seeded the enzyme-neutralized cell suspension into a 25 cm^2^ plastic culture flask in the presence of 6 mL of growth medium containing DMEM/F12 (1 : 1) medium supplemented with fetal calf serum (20%), horse serum (Gibco) (5%), penicillin (100 U/mL), amphotericin B (25 *μ*g/mL) and streptomycin (100 mg/mL; Cambrex, Verviers, Belgium), 2 mM L-glutamine (Cambrex), 0.1 mM nonessential amino acids (Gibco), 3 mM sodium pyruvate (Gibco), and bovine insulin (1 *μ*g/mL; USV, India). The cells were plated on plates precoated with 1% gelatin and incubated in 95% air and 5% CO_2_ at 37°C for ~2-3 h, to allow the differential attachment of fibroblast cells. The nonadhesive cells (LDSCs) were transferred into a sterile tube. Trypsin toxicity and cell viability of liver cells were assessed by trypan blue exclusion test as 85–90%. After counting, the LDSCs-enriched suspension was plated onto culture dishes at a density of 2 × 10^4^ cells per cm^2^. The cells were incubated in a humid 5% CO_2_ incubator at 37°C.

Selective removal of nonadherent population ensures the success of getting homogenous population and minimizes the contamination with cells of hematopoietic origin. The medium was replenished after 3 d which removes nonadherent cells and tissue debris and replaced with 5 mL of the maintenance medium. The maintenance medium was changed after every 48 h. When it reached confluent, the adherent cells were harvested by removing the medium and adding 2 mL of 0.25% trypsin/EDTA and passaged with a split ratio of 1 : 3. The culture medium was changed every 48 h and passaged twice per week at a split ratio of 1 : 4 or 1 : 3. The cells at passages 3–8 were used for* in vitro *experiments.

### 2.3. LDSCs Characterizations

#### 2.3.1. Immunophenotyping

Passage 4 LDSCs were harvested by trypsin digestion and the cells were centrifuged twice for 8 min, 300 ×g, at 4°C with 1 mL of cold PBS (range from 4 to 8°C). Then, resuspended 1 × 10^6^ cells were mixed with 100 *μ*L of cold PBS per Eppendorf tube and stain with anti-mouse or anti-goat EpCAM, Sca-1, CD29, CD44, CD105, GATA-4, CD34, CD45, and CD 90 for 1 h at 25°C and washed with cold PBS. The FITC-conjugated specific Ig antibodies and isotype antibodies were added, respectively, for 1 h at 25°C and washed with cold PBS. The concentrations of all primary antibodies were used in 1/200 dilution and 1/500 dilution of secondary antibodies was used for immunophenotype assays. The unstained cells were used as a control for all antibodies. The cells were washed again twice for 8 min, 300 ×g, at 4°C with 1 mL of cold PBS. Then paraformaldehyde (0.4%) was added to 200 *μ*L of cold PBS and analyzed by FACS analysis, after excluding the dead cells by forward/side scatter gating (2,00,000 events per sample by FACS Calibur).

### 2.4. Multilineage Differentiation and Assessment

#### 2.4.1. Differentiation of LDSCs to Osteogenic Lineage

Passage 4 LDSCs were harvested by trypsin digestion and the cells were seeded at a density of 5 × 10^3^/cm^2^ (or 1 × 10^4^ per well) in a 24-well plate with *α*-MEM supplemented with 10% (vol/vol) FBS, 10^−7^ M dexamethasone, 10 mM *β*-glycerol phosphate, and 50 *μ*M ascorbate-2-phosphate in a total volume of 500 *μ*L. Cells cultured in *α*-MEM supplemented with 10% (vol/vol) FBS were used as a negative control. The medium was replenished twice per week and the culture was maintained for 4 weeks. During culture maintenance, the cells were not passaged. After 4 weeks, mineralization was confirmed by alizarin red S staining.

#### 2.4.2. Assessment of Mineralization

Ca^2+^ mineralization of LDSCs induced to osteoblasts was assessed by Alizarin red biochemical staining, after 4 weeks of induction (with and without treatment for osteoblast for 4 weeks) [24]. The cells were rinsed with PBS and fixed with ice-cold 70% ethanol (Merck) for 1 h. The cells were washed twice with MQ H_2_O and stained with 50 mM AR-S (pH 4.2) at RT for 10 min. The cells were rinsed five times with MQ H_2_O succeeded by a 15 min wash with PBS with rotation to reduce nonspecific staining and the stained cells were photographed.

#### 2.4.3. Differentiation of LDSCs to Adipogenic Lineage

Passage 4 LDSCs were seeded into a 24-well culture plate at a density of 1 × 10^4^/cm^2^ (or 2 × 10^4^ per well) and incubated in *α*-MEM supplemented with 10% (vol/vol) FBS, 10^−6^ M dexamethasone, 0.5 *μ*M IBMX, and 10 ng/mL (wt/vol) insulin in a total volume of 500 *μ*L for 2 weeks. The cells were cultured in *α*-MEM supplemented with 10% (vol/vol) FBS as a negative control. Medium was changed twice per week and the culture was maintained for 2 weeks. Lipid accumulation was assessed biochemically by Oil Red O staining.

#### 2.4.4. Assessment of Lipid Accumulation

To detect adipogenic differentiation following induction, Oil Red O (ORO) staining was performed as previously reported by [25] to monitor lipid accumulation with slight modifications. The cells were rinsed with PBS and fixed in 4% paraformaldehyde for 1 hr at RT. Cells were washed once with DPBS and twice with MQ H_2_O. The cells were stained with 0.1% Oil Red O for 10 min at RT. The plates were washed with 60% of isopropanol to eliminate nonspecific staining and washed thrice with MQ H_2_O and the cells were photographed.

#### 2.4.5. Differentiation of LDSCs to Hepatocytes

Passage 4 LDSCs were seeded into a 24-well culture plate at a density of 1 × 10^4^/cm^2^ (or 2 × 10^4^ per well) in serum deprived condition for two days in DMEM supplemented with 20 ng/mL epidermal growth factor (EGF) and 10 ng/mL basic fibroblast growth factor (bFGF), prior to differentiation. The medium was replenished after 2 d with differentiation medium that consisted of DMEM supplemented with 20 ng/mL hepatocyte growth factor (HGF), 10 ng/mL bFGF, and 0.61 g/L nicotinamide for six days (medium change was done every 3 days). The differentiation medium was replaced with maturation medium which consisted of DMEM supplemented with 20 ng/mL oncostatin M, 1 *μ*mol/L dexamethasone (all from Sigma-Aldrich), and 50 mg/mL insulin. The medium was replenished twice per week and the culture was maintained for 3 weeks [26]. During cultural maintenance, the cells do not need to be passaged. Glycogen storage and albumin secretion were analyzed by periodic Acid-Schiff (PAS) staining and immunostaining, respectively.

#### 2.4.6. Assessment of Glycogen Storage

Glycogen storage of LDSCs culture was evaluated using PAS staining. The cells were fixed with 4% paraformaldehyde, then oxidized in 1% periodic acid for 5 min, and rinsed in dH_2_O. The cells were treated with Schiff's reagent for 15 min, and then color was developed in lukewarm dH_2_O for 5–10 min and assessed under a light microscope.

### 2.5. Immunocytochemistry

Cells were fixed with 4% paraformaldehyde and for 30 min. Permeabilization was done with 0.5% Triton X-100 in PBS for 15 min. After the washing steps (3 × 5 min) with PBS, cells were kept in blocking buffer 3% BSA for 1 h. The samples were then incubated with primary antibody Anti-BSA rabbit polyclonal IgG (07-248 Upstate Biotechnology) at a 1 : 200 dilution for overnight at 4°C. The cells were washed twice with PBS and incubated with secondary antibody mouse anti-rabbit IgG-FITC for 1 h at 1 : 200 at 37°C. Nuclei staining was performed using Hoechst 33258. Cells were examined by fluorescence microscope.

### 2.6. Gene Expression Analysis by RT-PCR

Furthermore, expression profile of hepatic stem cell-specific transcription factors, structural and functional proteins were analyzed by reverse transcriptase-polymerase chain reaction (RT-PCR). Total RNAs from liver-derived stem cell, neonatal liver (NL), and adult liver (AL) were isolated on primary culture by using Trizol method. RNA was converted to cDNA by using M-MLV Reverse Transcriptase (New England Biologicals, Beverly, MA) and was used as template for RT-PCR. The related PCR primers listed in Table 1 were used to produce the respective correlated products.

## 3. Results

### 3.1. Isolation of LDSCs

**Figure 1:**
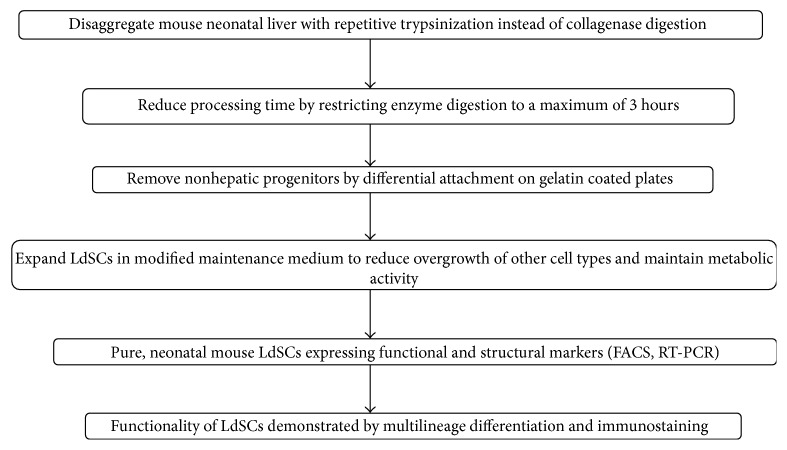
A schematic overview of the major steps followed in the isolation of LDSCs protocol.

**Figure 2:**
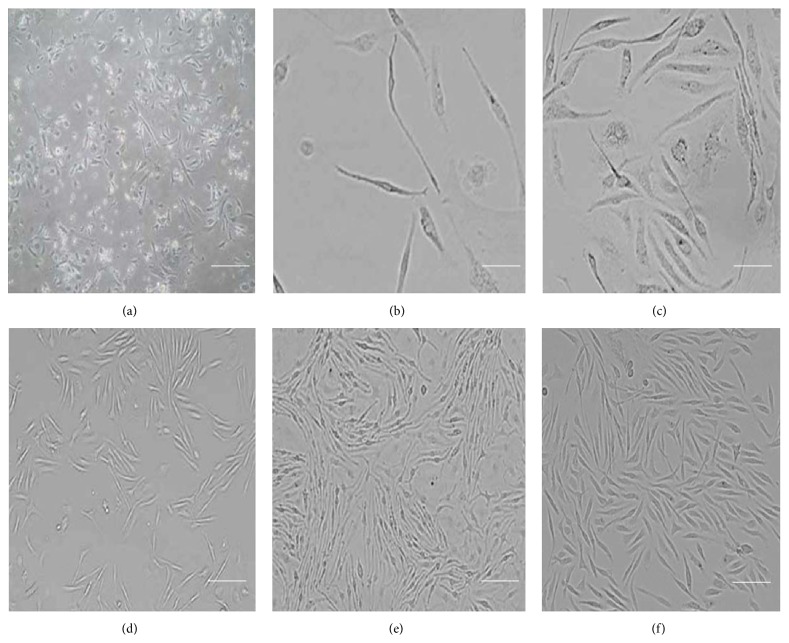
Morphological observation of liver-derived mouse stem cells. (a) LDSCs of different morphology were observed on 2nd day of culture (b, c) and after 3rd day elongated spindle shaped LDSCs were predominately observed from the initial culture. (d) LDSCs at their 60% confluency. (e) LDSCs at more than 90% confluent on day 6 of initial culture. (f) Passaged (P1) LDSCs at their 60% confluency. Scale bars represent 100 *μ*m (a, d, e, and f) and 50 *μ*m (a and b).

The aim of the current study is to derive stem cell populations from mouse neonatal liver using a simple and efficient protocol. LDSCs culture was isolated by digesting mouse neonatal liver with trypsin/EDTA for four minutes (Figure 1). The enzyme digested mixture was then seeded onto the flask already coated with 1% gelatin. Immediately after two to three hours, the culture medium along with the floating population of cells was transferred to another culture flask. Since most of all fibroblast-like cells adhere quickly to the gelatin coated culture flask, by changing to new culture flask it ensures the selective removal of fibroblasts. The reseeded suspension is mainly enriched with population of our interest, which adhered to the flask in 48 hours (Figure 2(a)). Further replenishing the adhered population with fresh media accelerated the growth and proliferation of the LDSC (Figures 2(b) and 2(c)) which was observed as increase in cell number inverted microscope (Nikon Eclipse TS100, Melville, NY).

An adherent population of spindle-shaped or vortex-shaped cells was observed after five days of culture (Figures 2(d) and 2(e)). The initial population of LDSC obtained after digestion was 0.3 × 10^6^/mm^2^ from every 100 mg of fetal liver tissue. Upon culture enrichment, the cell number of LDSC increased and reached confluency up to 0.2 × 10^6^/mm^2^ after 8 days (Figure 2(f)). After reaching confluency, the majority of the population morphologically appeared to be spindle-like cells. Briefly, after reaching confluency the cells were trypsinized, passaged, and maintained up to 4 weeks for further study.

### 3.2. Immunophenotyping of LDSCs

**Figure 3:**
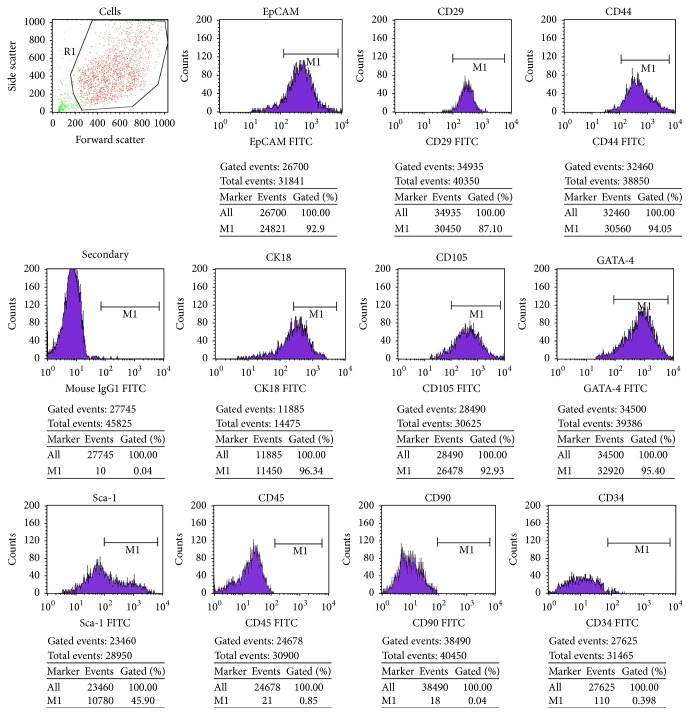
Immunophenotyping of LDSCs. Passaged 2 LDSCs were stained with fluorescein isothiocyanate (FITC) conjugated anti-mouse EpCAM, CD44, GATA-4, CD105, CD90, CD34, CK18 (Cytokeratin 18 (CK)), CD29, Sca-1, and CD45 along with its corresponding isotype control IgG1. FACS results showed that these cells were strong positive for liver stem cell markers EpCAM, GATA-4, CD44, CD105, CK18, and CD29 (*β*_1_ integrin) in addition to weak positive for Sca-1 and negative for hematopoietic markers CD45, CD34, and CD 90.

The cells were further characterized for their surface antigen by immunophenotyping. FACS analyses showed that majority of the populations were expressing markers like EpCAM, GATA-4, CD44, CD105, and CD29 (*β*_1_ integrin) more than 90% are suggesting strong positivity. In addition, LDSCs were found weak positive for Sca-1 and also negative for hematopoietic markers CD34, CD45, and CD 90 (Figure 3).

### 3.3. Multilineage Differentiation of LDSCs

**Figure 4:**
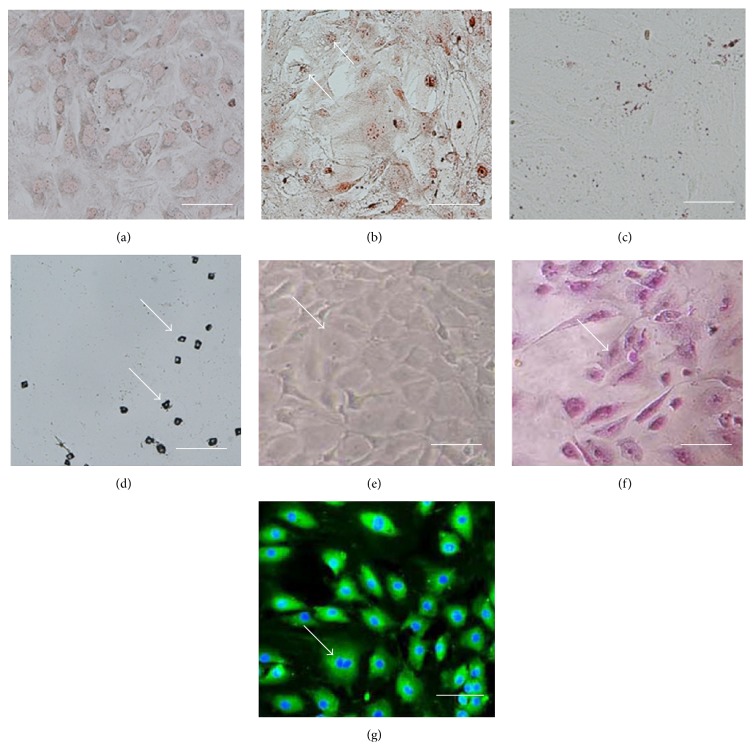
Differentiation potential of mouse liver-derived stem cells. (a, c) Uninduced LDSCs with their normal morphology stained negative for Oil Red O stain (a) and alizarin red (c). (b) Adipogenesis induced LDSCs were stained with Oil Red O stain showing positive for oil droplets after 4 weeks of induction. (d) Osteogenesis induced LDSCs were stained with alizarin red showing mineralized nodule after 4 weeks of induction. (e) Phase contrast image of hepatogenesis induced LDSCs showing typical polygonal shape from fibroblast shape. (f) Hepatogenesis was shown positive for PAS staining (glycogen storage). (g) Albumin expression in hepatocyte induced LdSCs showed by ICC. Scale bars represent 100 *μ*m (a)–(g).

LDSCs were induced to differentiate into lineages like osteoblasts, adipocytes, and hepatocytes. Passages 3–6 of LDSCs were used in the differentiation studies. Three weeks under the induction of osteogenic medium, LDSCs were differentiated into osteoblasts and was confirmed with alizarin red staining (Figures 4(c) and 4(d)) showed positive for the deposition of Ca^2+^ mineralization. Under adipogenic induction conditions for 3 weeks, the formation of intracellular microdroplets (accumulation of intracellular lipid droplets) was observed under microscope (Figure 4(b)) and it stained positive for Oil Red O (Figure 4(a)).

LDSCs showed hepatic differentiation by their phenotype from a spindle to a rather polygonal shape over a period of 36 days of culture, as observed by phase contrast microscope (Figure 4(g)). The presence of glycogen storage was also observed in differentiated hepatocyte by sensitive PAS staining (Figures 4(e) and 4(f)). Further, the functional potential of LDSC differentiated into hepatocytes was assessed by the liver specific marker of albumin secretion by immunocytochemistry and found to secrete albumin observed under phase fluorescent phase contrast microscope.

### 3.4. Gene Expression Profiling of LDSCs

**Figure 5:**
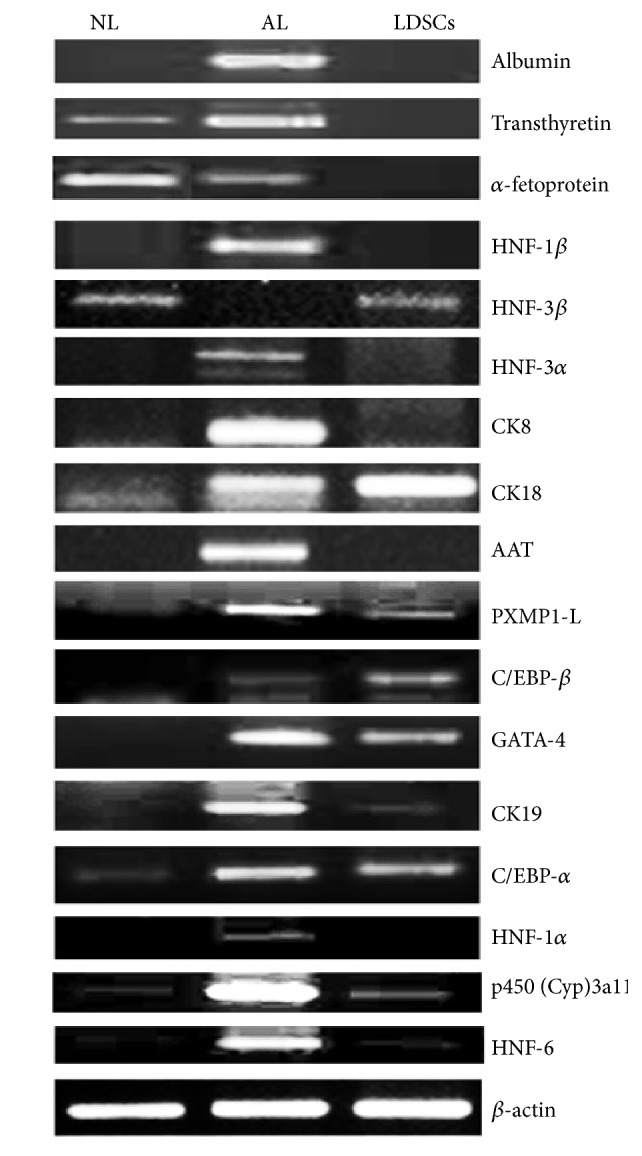
Hepatic markers and their transcription factor mRNA expression by RT-PCR. The RNA was isolated from LDSCs and the mRNA expressions were compared with neonatal liver (NL) and adult liver (AL) for albumin, transthyretin, AAT, AFP, CK8, CK18, CK19, p450 (Cyp)3a11, PXMP1-L HNF-1*α*, HNF-1*β*, HNF-3*α*, HNF-3*β*, HNF-4*α*, HNF-6, GATA-4, C/EBP-*α*, and C/EBP-*β*. LDSCs show the mRNA expression for HNF-3*β*, CK18, PXMP1-L, C/EBP-*α*, C/EBP-*β* GATA-4, CK18, p450 (Cyp)3a11, and HNF-6, negative for hepatic markers.

The expression profile of liver-derived stem cell markers was screened for the presence of liver stem cell markers using PCR analysis. Data from the PCR analysis showed that LDSC expressed transcription factors (HNF-3*β*, HNF-6, C/EBP-*β*, C/EBP-*α*, and GATA-4) and also structural and functional proteins (CK18, CK19, p450 Cyp3a11, and PXMP1-L) of hepatic stem cells (Figure 5). Notably, the functional proteins mRNA expressions were negative (albumin, transthyretin, *α*-fetoprotein, and AAT) in case of LdSCs, whereas transthyretin and *α*-fetoprotein were found expressed in neonatal liver (Table 2).

## 4. Discussion

The current study involved in the derivation of LDSCs from neonatal mice liver suggested that, when compared to adult mice liver, the neonatal mouse has a higher number of stem cells with high proliferation and differentiation potential [27]. LDSCs from neonatal liver possess features including spindle shape-like morphology, adherent to the plastic surface, devoid of hematopoietic markers, and possess multilineage differentiation potential into osteoblasts, adipocyte, and hepatocytes similar to MSCs [28]. Flow cytometric analysis showed that the cells were positive for EpCAM (CD326), GATA-4, CD105 (endoglin), and mesenchymal cells markers including CD29 (*β*_1_ integrin) and CD44 (receptor for hyaluronate and osteopontin), in addition to being weak positive for Sca-1 (a murine hematopoietic stem cell and MSC marker). LDSCs were negative for hematopoietic markers such as CD34, CD45 (primitive hematopoietic progenitor and endothelial cell marker), and CD90 (GPI-anchored glycoprotein), which are congruent with their counterparts from human and murine. The cell surface antigen profile showed that the cultured LDSCs belong to a population of nonhematopoietic, nonendothelial, slightly mesenchyme origin, and it behaves similar to adult mouse hepatic stem cells under* in vitro* culture conditions. LDSCs are capable of self-renewal and are multipotent, able to give rise to committed biliary progenitors and hepatocyte lineages. Hepatic lineages were identified by morphological changes and stained with periodic Acid-Schiff (PAS) for glycogen storage and assessment of albumin secretion [29] by ICC as well as by another multilineage differentiation to osteoblasts and adipocytes (Figure 4).

The expression profiling showed the specific markers for transcriptional and structural proteins of LDSCs, with no expression of mature liver functional markers [10]. These findings suggested that the isolated cells resembled liver progenitors cells; however, they lack the mature hepatocyte marker like albumin and so forth. The reason for expressing the mesenchymal counter parts may be due to interaction of committed endodermal cells with mesenchymal components of the primitive liver during embryogenesis.

In the current study, LDSCs were efficiently isolated by a shortened protocol that limited the usage of enzyme cocktails like collagenase and hyaluronidase and also with minimal exposure to enzyme digestion time. This study followed a modified protocol as reported earlier by [30, 31] where 1% gelatin has been used to coat culture dishes, which aids in selective removal of fibroblasts due to its higher affinity to a collagenous extracellular matrix like gelatin [32]. In our study we used one-step enrichment procedure followed by enzyme digestion that effectively removes fibroblasts and improves culture homogeneity. The culture conditions were optimized for DMEM/F12 which includes supplementation of insulin, sodium pyruvate, glutamine, nonessential amino acids, and horse serum were supported the LDSCs in stimulating the glycolysis, and preventing accumulation of metabolic end products like lactate, and reduces the overgrowth of epithelial and fibroblasts like-cells [16, 33] as compared to the maintenance medium M199, which was used by earlier workers [30, 34–36].

## 5. Conclusion

Current study describes a rapid, reproducible, and efficient protocol for isolation of homogenous population of LDSCs. These cells have potential to become functional hepatocytes. Further, LDSCs can be used as* in vitro *model system for assessing various drug toxicity assays and preclinical trials in pharmacokinetic studies and in various liver based tissue engineering approaches.

## Figures and Tables

**Table 1:** List of primers used in gene expression profiling.

Genes	Primer sequence (5′-3′)	Tm	Length
HNF-1*α*	FP: 5′-CGAAGATGGTCAAGTCGTAC-3′ RP: 5′-GGCAAACCAGTTGTAGACAC-3′	55°C	500 bp

HNF-1*β*	FP: 5′-TTCAGTCAACAGAACCAGGG-3′ RP: 5′-CTCTGTGCAATGGCCATGAC-3′	57.2°C	721 bp

HNF-3*α*	FP: 5′-CATGAGAGCAACGACTGGAA-3′ RP: 5′-TTGGCGTAGGACATGTTGAA-3′	55.2°C	182 bp

HNF-3*β*	FP: 5′-AGAGGACTGAGGTAACTGAC-3′ RP: 5′-GACTCGGACTCAGGTGAGGT-3′	60.2°C	415 bp

HNF-6	FP: 5′-CAGCGTATCACCACCGAGCT-5′ RP: 5′-CTCTGTCCTTCCCATGTTCT-3′	55°C	250 bp

C/EBP-*α*	FP: 5′-TGGACAAGAACAGCAACGAG-3′ RP: 5′-TCACTGGTCAACTCCAGCAC-3′	56°C	126 bp

C/EBP-*β*	FP: 5′-GAGCGACGAGTACAAGATGCG-3′ RP: 5′-TTGTGCTGCGTCTCCAGGTTG-3′	61°C	95 bp

GATA-4	FP: 5′-CTGTCATCTCACTATGGGCA-3′ RP: 5′-CAAGTCCGAGCAGGAATTTG-3′	59°C	257 bp

CK8	FP: 5′-ATCGAGATCACCACCTACCG-3′ RP: 5′-TGAAGCCAGGGCTAGTGAGT-3′	55°C	127 bp

CK18	FP: 5′-CGAGGCACTCAAGGAAGAAC-3′ RP: 5′-GCTGAGGTCCTGAGATTTGG-3′	57°C	130 bp

CK19	FP: 5′-ACCCTCCCGAGATTACAACC-3′ RP: 5′-CAAGGCGTGTTCTGTCTCAA-3′	58°C	160 bp

p450 Cyp3a11	FP: 5′-TGAGGCAGAAGGCAAAGAAA-3′ RP: 5′-GGTATTCCATCTCCATCACA-3′	55°C	590 bp

PXMP1-L	FP: 5′-CTTCAGACCCAGAGAGAGCTG-3′ RP: 5′-CCCGTGTTGCCTGTGATGAGC-3′	62°C	475 bp

Albumin	FP: 5′-TGAACTGGCTGACTGCTGTG-3′ RP: 5′-CATCCTTGGCCTCAGCATAG-3′	57°C	718 bp

Transthyretin	FP: 5′-AGTCCTGGATGCTGTCCGAG-3′ RP: 5′-TTCCTGAGCTGCTAACACGG-3′	62°C	440 bp

AFP	FP: 5′-TCGTATTCCAACAGGAGG-3′ RP: 5′-AGGCTTTTGCTTCACCAG-3′	55°C	174 bp

AAT	FP: 5′-AATGGAAGAAGCCATTCGAT-3′ RP: 5′-AAGACTGTAACTGCTGCAGC-3′	57.2°C	484 bp

*β*-actin (internal control)	FP: 5′-TTCCTTCTTGGGTATGGAAT-3′ RP: 5′-GAGCAATGATCTTGATCTTC-3′	55°C	206 bp

**Table 2:** Summary of the phenotype and genotype of isolated LDSCs.

FACS	
Liver marker	Expression

EpCAM	+++
GATA-4	+++
CK18	+++

Mesenchyme marker	

CD105	+++
CD29	+++
CD44	+++
Sca-1	++

Hematopoietic marker	

CD34	−
CD45	−
CD90	−

RT-PCR	

Transcriptional markers	Expression

HNF-3*β*	++
HNF-6	+
C/EBP-*α*	++
C/EBP-*β*	++
HNF-1*α*	−
HNF-1*β*	−
HNF-3*α*	−
GATA-4	++

Structural markers	

CK8	−
CK18	++++
CK19	+

Functional markers	

Albumin	−
Transthyretin	−
*α*-fetoprotein	−
AAT	−
p450 Cyp3a11	++
PXMP1-L	+

Percentage of expression: ++++: 95–100%, +++: 75–95%, ++: 40–75%, +: 10–35%, and −: 0-1%.

## References

[1] Hixson D. C., Faris R. A., Yang L., Novikoff P., Sirica A. E. (1992). Antigenic clues to liver development, renewal, and carcinogenesis: an integrated model. *The Role of Cell Types in Hepatocarcinogenesis*.

[2] Saxena R., Theise N. D., Crawford J. M. (1999). Microanatomy of the human liver—exploring the hidden interfaces. *Hepatology*.

[3] Solt D. B., Medline A., Farber E. (1977). Rapid emergence of carcinogen-induced hyperplastic lesions in a new model for the sequential analysis of liver carcinogenesis. *The American Journal of Pathology*.

[4] Evarts R. P., Hu Z., Omori N., Omori M., Marsden E. R., Thorgeirsson S. S. (1996). Precursor-product relationship between oval cells and hepatocytes: comparison between tritiated thymidine and bromodeoxyuridine as tracers. *Carcinogenesis*.

[5] Lázaro C. A., Rhim J. A., Yamada Y., Fausto N. (1998). Generation of hepatocytes from oval cell precursors in culture. *Cancer Research*.

[6] Kubota H., Reid L. M. (2000). Clonogenic hepatoblasts, common precursors for hepatocytic and biliary lineages, are lacking classical major histocompatibility complex class I antigen. *Proceedings of the National Academy of Sciences of the United States of America*.

[7] Mahieu-Caputo D., Allain J.-E., Branger J. (2004). Repopulation of athymic mouse liver by cryopreserved early human fetal hepatoblasts. *Human Gene Therapy*.

[8] Lázaro C. A., Croager E. J., Mitchell C. (2003). Establishment; characterization; and long-term maintenance of cultures of human fetal hepatocytes. *Hepatology*.

[9] Dan Y. Y., Riehle K. J., Lazaro C. (2006). Isolation of multipotent progenitor cells from human fetal liver capable of differentiating into liver and mesenchymal lineages. *Proceedings of the National Academy of Sciences of the United States of America*.

[10] Schmelzer E., Wauthier E., Reid L. M. (2006). The phenotypes of pluripotent human hepatic progenitors. *Stem Cells*.

[11] Schmelzer E., Zhang L., Bruce A. (2007). Human hepatic stem cells from fetal and postnatal donors. *Journal of Experimental Medicine*.

[12] Turner W. S., Schmelzer E., McClelland R., Wauthier E., Chen W., Reid L. M. (2007). Human hepatoblast phenotype maintained by hyaluronan hydrogels. *Journal of Biomedical Materials Research—Part B Applied Biomaterials*.

[13] Wang J., Clark J. B., Rhee G.-S., Fair J. H., Reid L. M., Gerber D. A. (2003). Proliferation and hepatic differentiation of adult-derived progenitor cells. *Cells Tissues Organs*.

[14] Fougère-Deschatrette C., Imaizumi-Scherrer T., Strick-Marchand H. (2006). Plasticity of hepatic cell differentiation: bipotential adult mouse liver clonal cell lines competent to differentiate in vitro and in vivo. *Stem Cells*.

[15] Fujikawa T., Hirose T., Fujii H. (2003). Purification of adult hepatic progenitor cells using green fluorescent protein GFP-transgenic mice and fluorescence-activated cell sorting. *Journal of Hepatology*.

[16] Avital I., Inderbitzin D., Aoki T. (2001). Isolation, characterization, and transplantation of bone marrow-derived hepatocyte stem cells. *Biochemical and Biophysical Research Communications*.

[17] Shupe T. D., Piscaglia A. C., Oh S.-H., Gasbarrini A., Petersen B. E. (2009). Isolation and characterization of hepatic stem cells, or ‘oval cells,’ from rat livers. *Methods in Molecular Biology*.

[18] Sreejit P., Kumar S., Verma R. S. (2008). An improved protocol for primary culture of cardiomyocyte from neonatal mice. *In Vitro Cellular and Developmental Biology: Animal*.

[19] Lilja H., Arkadopoulos N., Blanc P. (1997). Fetal rat hepatocytes: isolation, characterization, and transplantation in the nagase analbuminemic rats. *Transplantation*.

[20] Lilja H. P., Blanc P., Demetriou A. A., Rozga J. (1998). Response of cultured fetal and adult rat hepatocytes to growth factors and cyclosporine. *Cell Transplantation*.

[21] Tanimizu N., Nishikawa M., Saito H., Tsujimura T., Miyajima A. (2003). Isolation of hepatoblasts based on the expression of Dlk/Pref-1. *Journal of Cell Science*.

[22] Lee D., Lee K. (2014). Hepatocyte isolation, culture and its clinical applications. *Hanyang Medical Reviews*.

[23] Howard R. B., Christensen A. K., Gibbs F. A., Pesch L. A. (1967). The enzymatic preparation of isolated intact parenchymal cells from rat liver. *Journal of Cell Biology*.

[24] Stanford C. M., Jacobson P. A., Eanes E. D., Lembke L. A., Midura R. J. (1995). Rapidly forming apatitic mineral in an osteoblastic cell line (UMR 106-01 BSP). *The Journal of Biological Chemistry*.

[25] Ramírez-Zacarías J. L., Castro-Muñozledo F., Kuri-Harcuch W. (1992). Quantitation of adipose conversion and triglycerides by staining intracytoplasmic lipids with oil red O. *Histochemistry*.

[26] Lee K.-D., Kuo T. K.-C., Whang-Peng J. (2004). In vitro hepatic differentiation of human mesenchymal stem cells. *Hepatology*.

[27] Fiegel H. C., Lange C., Kneser U. (2006). Fetal and adult liver stem cells for liver regeneration and tissue engineering. *Journal of Cellular and Molecular Medicine*.

[28] Dominici M., Le Blanc K., Mueller I. (2006). Minimal criteria for defining multipotent mesenchymal stromal cells. The International Society for Cellular Therapy position statement. *Cytotherapy*.

[29] Schudt C. (1979). Regulation of glycogen synthesis in rat-hepatocyte cultures by glucose, insulin and glucocorticoids. *European Journal of Biochemistry*.

[30] Song W., Lu X., Feng Q. (2000). Tumor necrosis factor-*α* induces apoptosis via inducible nitric oxide synthase in neonatal mouse cardiomyocytes. *Cardiovascular Research*.

[31] Matsuura K., Wada H., Nagai T. (2004). Cardiomyocytes fuse with surrounding noncardiomyocytes and reenter the cell cycle. *Journal of Cell Biology*.

[32] Haas R., Banerji S. S., Culp L. A. (1984). Adhesion site composition of murine fibroblasts cultured on gelatin-coated substrata. *Journal of Cellular Physiology*.

[33] Fioramonti M. C., Bryant J. C., Mcquilkin W. T., Evans V. J., Sanford K. K., Earle W. R. (1955). The effect of horse serum residue and chemically defined supplements on proliferation of strain L clone 929 cells from the mouse. *Cancer Research*.

[34] Simpson P., Savion S. (1982). Differentiation of myocytes in single cell cultures with and without proliferating nonmyocardial cells. *Circulation Research*.

[35] Nickson P., Toth A., Erhardt P. (2007). PUMA is critical for neonatal cardiomyocyte apoptosis induced by endoplasmic reticulum stress. *Cardiovascular Research*.

[36] Bryja V., Bonilla S., Čajánek L. (2006). An efficient method for the derivation of mouse embryonic stem cells. *Stem Cells*.

